# Correction of sequence-dependent ambiguous bases (Ns) from the 454 pyrosequencing system

**DOI:** 10.1093/nar/gku070

**Published:** 2014-01-23

**Authors:** Sunguk Shin, Joonhong Park

**Affiliations:** School of Civil and Environmental Engineering and WCU Center for Green Metagenomics, Yonsei University, Shinchon-dong 134, Seodaemoon-gu, Seoul, Republic of Korea

## Abstract

Pyrosequencing of the 16S ribosomal RNA gene (16S) has become one of the most popular methods to assess microbial diversity. Pyrosequencing reads containing ambiguous bases (Ns) are generally discarded based on the assumptions of their non-sequence-dependent formation and high error rates. However, taxonomic composition differed by removal of reads with Ns. We determined whether Ns from pyrosequencing occur in a sequence-dependent manner. Our reads and the corresponding flow value data revealed occurrence of sequence-specific N errors with a common sequential pattern (a homopolymer + a few nucleotides with bases other than the homopolymer + N) and revealed that the nucleotide base of the homopolymer is the true base for the following N. Using an algorithm reflecting this sequence-dependent pattern, we corrected the Ns in the 16S (86.54%), *bphD* (81.37%) and *nifH* (81.55%) amplicon reads from a mock community with high precisions of 95.4, 96.9 and 100%, respectively. The new N correction method was applicable for determining most of Ns in amplicon reads from a soil sample, resulting in reducing taxonomic biases associated with N errors and in shotgun sequencing reads from public metagenome data. The method improves the accuracy and precision of microbial community analysis and genome sequencing using 454 pyrosequencing.

## INTRODUCTION

Massively parallel 454 pyrosequencing is a recently developed popular method (1) to more extensively assess the molecular diversity and taxonomy of microbial communities in the environment (2,3) or human microbiome samples (4) without cultivation. In addition to amplicon sequencing, pyrosequencing is one of the most popular methods for genome sequencing because the 454 pyrosequencing system is cost- and time-efficient. However, its primary drawback is sequencing errors. Many scientists are interested in pyrosequencing errors because they may overestimate microbial diversity (5). Errors are typically classified into four types: insertion, deletion, mismatch and ambiguous base, N (6). The 454 pyrosequencing system and traditional Sanger sequencing have different error formation patterns (7). Light intensity is proportional to the length of homopolymeric bases and is converted into flow values in the 454 pyrosequencing system. However, flow values, particularly from long homopolymers, can be imprecise and result in higher homopolymer insertion/deletion error rates and lower substitution error rates for the pyrosequencing system than the traditional Sanger method (1). Many methods, such as the PyroNoise method (8), have been developed to accurately determine homopolymer lengths.

N is a significantly important source of error in 454 pyrosequencing. The number of N errors may represent up to 21% of all errors (6). Interestingly, the number of Ns was reported to correlate with the number of other types of errors, suggesting the possible association of Ns with insertions, deletions and substitutions (6). In the majority of studies about pyrosequencing errors, simple removal of the reads with Ns is used to reduce the overall sequencing error rates (5,9). The basic assumption underlying the simple removal of N-containing reads is that multi-templated beads and/or the non-synchronized extension of fragments occurs in a random manner and has effects on N-containing reads. However, this assumption has yet to be justified. N formation rates are dramatically different among studies (6,10) and might be sequence-dependent. If N formation is dependent on the flanking sequence or specific sequence motifs, a specific group of sequence reads would be selectively removed, possibly resulting in a biased loss of microbial community sequences, which may affect the relative quantification of certain microbial populations. However, flanking sequence dependence and/or the existence of specific sequence motifs has yet to be identified in the 454 pyrosequencing system.

We hypothesized that homopolymers directly affect N formation in the 454 pyrosequencing system because long homopolymers affect the signals of downstream flanking nucleotides in the old pyrosequencing system (11). Therefore, the Ns would be particularly associated with homopolymers and their insertion/deletion errors, and the occurrence of Ns would be sequence-specific rather than random. In this study, the possibility of flanking sequence-dependent N errors in the 454 pyrosequencing system was directly examined with bacterial gene amplicon reads from both defined pure cultures and a mock community. We explored the possible existence of sequence motifs around the Ns of amplicon reads from the 16S V1–V3 region and two functional gene regions, the 2-hydroxy-6-oxo-6-phenylhexa-2,4-dienoate hydrolase gene (*bphD*) and the nitrogen fixation gene (*nifH*). 16S was chosen because it is the standard molecular marker (12) for microbial identification and diversity estimation (3), and *bphD* and *nifH* were chosen because they are representative genes for the microbial degradation of biphenyl/polychlorinated biphenyls (BP/PCBs) (13) and bacterial nitrogen fixation (14), respectively. Based on the findings from the sequence motifs related to N errors, we developed a new method to correct N errors in pyrosequenced amplicons from environmental samples and validated the method with public metagenomic data.

## MATERIALS AND METHODS

For defined pure bacterial cultures, *Staphylococcus epidermidis* ATCC 12228 (15), *Roseobacter denitrificans* OCh 114 (16), *Rhodococcus* sp. RHA1 (17) and *Polaromonas naphthalenivorans* CJ2 (18) were used. Barcoded DNAs were pooled for one sequencing run. Defined mock community DNA was constructed from the genomes of the following 20 bacterial isolates, which have been genome sequenced except *Ralstonia pickettii* PKO1 (19): *Xanthomonas campestris* ATCC 33913, *Sphingobium yanoikuyae* B1, *Nostoc* sp. PCC 7120, *Bacillus cereus* ATCC 14579, *Chromobacterium violaceum* ATCC 12472, *S. epidermidis* ATCC 12228, *Corynebacterium glutamicum* ATCC 13032, *Rhodospirillum rubrum* ATCC 11170, *Burkholderia xenovorans* LB400, *R. denitrificans* OCh 114, *Rhodococcus* sp. RHA1, *P. naphthalenivorans* CJ2, *Burkholderia vietnamiensis* G4, *Pseudomonas putida* F1, *Ochrobactrum anthropi* ATCC 49188^T^, *Ralstonia pickettii* PKO1, *Desulfitobacterium hafniense* DCB-2, *Rhodobacter sphaeroides* KD131, *Escherichia coli* K12 substr. W3110 and *Neisseria sicca* ATCC 29256. The same amount of genomic DNA was extracted from each microbe; however, the copy numbers of 16S, *bphD* and *nifH* differed among the microbes. In addition, the microbial genomes varied in size (Supplementary Table S1). For an environmental sample, the direct extraction of DNA from soil from Muak Mountain, Seoul, Korea, was used. The DNAs from the bacterial pure cultures, mock community and soil microbial community were extracted using a PowerSoil DNA isolation kit (MoBio Laboratories, Inc., Carlsbad, CA, USA) and sequenced in independent runs. For public pyrosequencing data, the sequence read archive data (accession: SRX015617) of 10 reference organisms (20) were downloaded from NCBI.

### Gene-targeted (amplicon) pyrosequencing

The regions of the 16S V1–V3, 16S V4–V5, *bphD* and *nifH* were amplified using the corresponding primers with barcodes and adaptors (19). All polymerase chain reaction (PCR) amplifications were performed with AccuPrime™ Taq DNA Polymerase High Fidelity, and the PCR products were purified with a QIAquick PCR purification kit (Qiagen, Valencia, CA, USA). After quantification with a NanoDrop ND-1000 (Thermo Scientific, DE, USA), each mixture of PCR products was purified and concentrated with a MinElute PCR purification kit (Qiagen, Valencia, CA, USA). Titanium pyrosequencing of the PCR products was performed on a GS-FLX system with GS FLX Titanium Sequencing Kit XL + and GS FLX Titanium PicoTiterPlate Kit 70 × 75 at Macrogen (Seoul, South Korea). Briefly, after denaturation of the double-stranded DNA fragments of the PCR products, the single-stranded DNA fragments were mixed with capture beads. The emulsion PCR reagents and oil were then added. Emulsion PCR was performed according to the manufacturer’s instructions (Roche, Nutley, NJ, USA), and the capture beads with DNA fragments were isolated and loaded into a PicoTiterPlate™. The four nucleotide reagents flowed sequentially, and the light intensity was detected with charge-coupled device sensors.

### Computational work

MUSCLE (21) was used to align the reads as shown in our workflow (Supplementary Figure S2) with an optimized MUSCLE parameter setting (-maxiter 2). We used Jalview (22) to detect contamination and calculate consensus of sequence motifs of N formation among the aligned reads (Supplementary Figure S3). BLAST (23) was used to remove sequence contamination. The settings for our length filtering were >300 bp, >300 bp, >300 bp and >200 bp for 16S V1–V3, 16S V4–V5, *bphD* and *nifH*, respectively. Reads from the pyrosequencing system were matched with their reference sequences. The Ribosomal Database Project Classifier (24) was used to remove 16S region sequence contamination in the mock community with an 80% confidence threshold for classification to root and to analyze any change of microbial community structure. The numbers of errors were determined by BLAST analysis with an optimized BLAST parameter setting (-max_target_seqs 1) to match the only one reference sequence. In addition, we deleted the primer areas because damaged primers can cause sequencing errors and the formation of Ns (Supplementary Table S4). To construct the reference sequences, the genomic sequences for 16S, *bphD* and *nifH* genes from NCBI were downloaded and matched with our primer sets. To discard unmatched sequences, all downloaded sequences were tested using a hidden Markov model. A few dominant sequences from our sequencing data were added as reference sequences.

## RESULTS

### Determination of sequence motifs

The N error rates differed significantly depending on the sequencing regions (16S versus *bphD* versus *nifH*) and organisms (Table 1). If the formation of Ns is a random event independent of the flanking sequence, the occurrence of Ns should be evenly distributed along the reads. Even if the non-synchronized extension of fragments is the primary reason for N errors, N errors should occur more densely after certain positions. However, the N error rates were significantly increased at certain positions, and their distribution varied depending on both the gene regions but also on organisms (Figure 1). Kurtosis is a measure of the peakedness of a distribution (25), and the excess kurtosis values are obtained by subtracting three from the kurtosis values (Table 1). The widely varying kurtosis values support that the N error distributions varied depending on gene regions as well as organisms. The PCR products of the *bphD* region from *P. naphthalenivorans* CJ2 (Polaromonas_bphD) had the highest N error rate with the longest homopolymers, GGGGG (Table 2). The N error rate tended to increase with the length of the upstream homopolymer.

**Figure 1.**
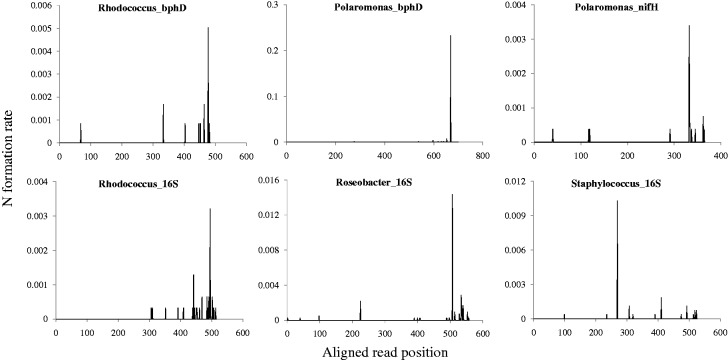
Distributions of N errors in the aligned reads of pure cultures including *Rhodococcus* sp. RHA1 *bphD* gene region (Rhodococcus_bphD), *P. naphthalenivorans* CJ2 *bphD* gene region (Polaromonas_bphD), *P. naphthalenivorans* CJ2 *nifH* gene region (Polaromonas_nifH), *Rhodococcus* sp. RHA1 16S V1–V3 region (Rhodococcus_16S), *R. denitrificans* OCh 114 16S V1–V3 region (Roseobacter_16S) and *S. epidermidis* ATCC 12228 16S V1–V3 region (Staphylococcus_16S). Genomic DNAs of the pure cultures were separately barcoded and amplified, but they were pooled for one sequencing run. The Y-axis unit is N error rate per base.

**Table 1.** N error rates from the pure bacterial cultures used in this study

PCR products	Number of reads (per read)	N error rate (per error^a^)	N error rate value	Excess kurtosis
Staphylococcus_16S	2723	0.0180	0.0309	−0.24
Roseobacter_16S	4178	0.0311	0.0208	6.96
Rhodococcus_16S	3113	0.0128	0.0082	3.11
Polaromonas_bphD	1645	0.2657	0.1566	68.74
Rhodococcus_bphD	1194	0.0126	0.0158	8.14
Polaromonas_nifH	2648	0.0019	0.0062	3.32

^a^Error includes insertion, deletion, mismatch and N errors.

**Table 2.** Sequence motifs of the N errors from different pure cultures and genes

Organism_gene	Dominant base at N (true base)	Sequence motif (per read)	N error rate
Staphylococcus_16S	C	CACCCTNTCAGGT	0.0103
Roseobacter_16S	C	CCCCGGNTAACTC	0.0144
Polaromonas_bphD	G	GGGGGTNTGTTCG	0.2328
Rhodococcus_bphD	T	ACCTTCNCGG	0.0050

The underlined letters denote the homopolymers in front of the Ns.

#### Sequential pattern of N errors from pure cultures

Careful inspection of the sequence reads around the positions of the dense N errors revealed sequence motifs in front of the Ns. In particular, each sequence motif consisted of a homopolymer and 1–2 bp nucleotides with bases other than the upstream homopolymer in front of the N. Furthermore, the dominant bases at the N positions in the aligned sequences were the same bases as the homopolymers in front of the Ns. For example, in ‘Staphylococcus_16S’, the dominant base at the N position is cytosine, the same base as in the upstream homopolymer.

#### Flow values around Ns

The sequential pattern was confirmed by analyzing the flow values around the Ns. The same pattern was observed in the other organisms and gene regions, with flow values of ∼0.5 (Table 3). The most likely reason that the flow value for the N position was <1 is that the formation of excessive by-products from the homopolymer synthesis prohibited reactions for light emission. It is also possible that a nucleotide reagent is insufficient because of overuse of the same nucleotide reagent for homopolymer detection in the previous cycle. In any case, the upstream homopolymer affected the flow value only in the first downstream cycle but not in the later cycles, as for ‘Staphylococcus_16S’, in which the flow value of the last cytosine nucleotide was recovered almost to an integer (1.10).

**Table 3.** Analysis of the flow values of the nucleotides around Ns

Staphylococcus_16S											
Nucleotide	A	C	G	T	A	**C**	G	T	A	C	G
Average	1.1	3.04	0.18	1.02	0.02	**0.46**	0.14	0.92	0.07	1.1	0.08
Standard deviation	0.083	0.128	0.066	0.081	0.034	**0.038**	0.055	0.097	0.048	0.085	0.065

Roseobacter_16S											
Nucleotide	A	C	G	T	A	**C**	G	T	A	C	G
Average	2.77	4.01	2.08	0.2	0.14	**0.44**	0.13	0.96	2.26	0.19	0.25
Standard deviation	0.14	0.159	0.146	0.098	0.12	**0.05**	0.087	0.124	0.134	0.168	0.104

Polaromonas_bphD											
Nucleotide	C	G	T	A	C	**G**	T	A	C	G	T
Average	0.2	4.87	0.99	0.12	0.19	**0.44**	0.98	0.13	0.12	1.26	2
Standard deviation	0.04	0.198	0.099	0.028	0.066	**0.052**	0.129	0.033	0.086	0.097	0.103

Rhodococcus_bphD											
Nucleotide	G	T	A	C	G	**T**	A	C	G	T	A
Average	0.12	2.17	0.1	0.73	0.32	**0.43**	0.28	1.05	1.86	0.23	0.18
Standard deviation	0.031	0.059	0.026	0.069	0.087	**0.077**	0.021	0.009	0.039	0.016	0.107

The bold letters are the flow values of the Ns. The underlined letters denote the flow values of the homopolymers in front of the Ns.

Ns are hypothesized to be formed by multi-templated beads and/or non-synchronized extension of fragments (6). However, in addition to the flowgram analysis, further examination of the consensus of aligned reads containing Ns showed a single N error at one position of aligned reads, ruling out the possibility of multiple N occurrence due to multi-templated beads and/or non-synchronized extension of fragments in the areas of high N formation frequency (an example is shown in Supplementary Figure S3).

### N correction algorithm development

Based on the results of the sequence motif study, a new algorithm for N correction was developed (Figure 2). Briefly, the N correction algorithm identifies upstream homopolymers (≧2 bp) in front of the Ns and then replaces the Ns with the same bases of the upstream homopolymers. Between N and its upstream homopolymer, there are 1–3 base(s) other than the upstream homopolymer. In this algorithm, we assume that the upstream homopolymer temporally affects only the same base of the first downstream cycle and not the subsequent cycles. The Ns were corrected in the following order: (i) a different base existing between N and the upstream homopolymer, (ii) two different bases existing between N and the upstream homopolymer and (iii) three different bases existing between N and the upstream homopolymer. The algorithm was coded in Perl (Supplementary Script S5), and our N correction program is available for Windows (Supplementary Program S10).

**Figure 2.**
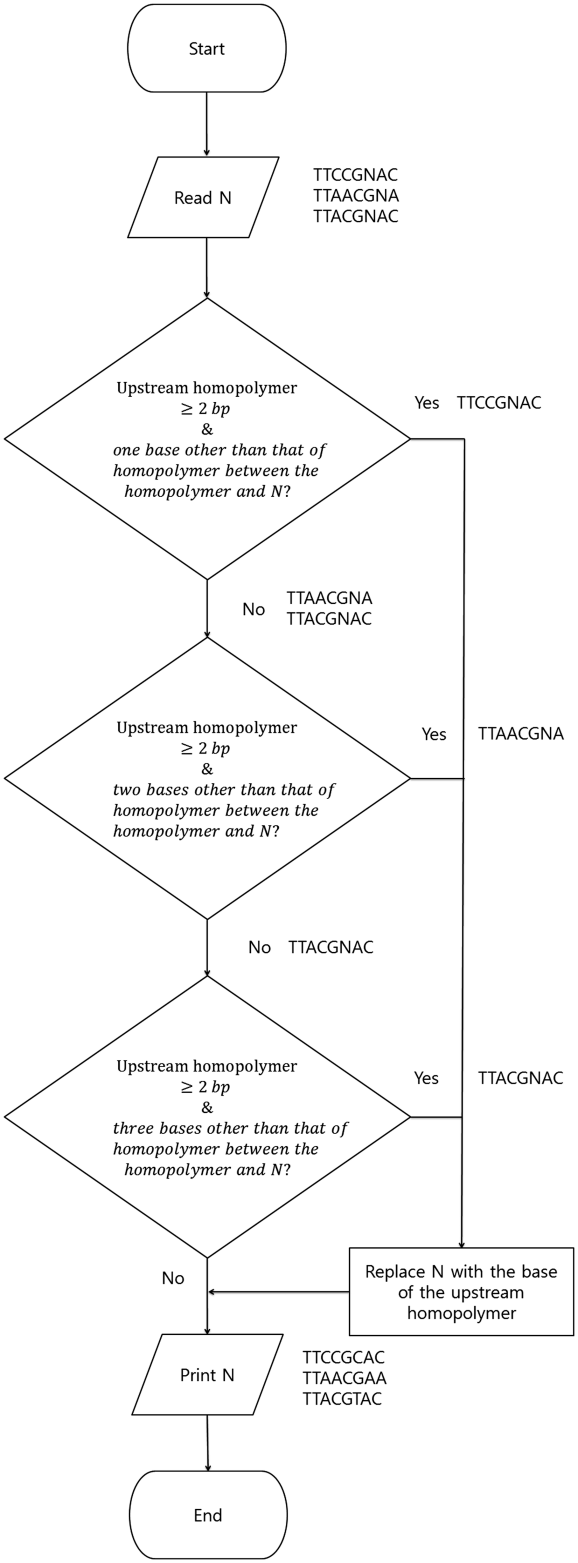
Flow chart for the N correction algorithm developed in this study. TTCCGNAC, TTAACGNA and TTACGNAC are examples of DNA sequences to explain how the flow chart works.

### Validation of the N correction method

For validation of the developed N correction method, N correction performance and precision were evaluated using the defined mock community and its reference sequences. Compared with pure cultures (Figure 1), the N errors of the mock community were more widely distributed through its corresponding sequence region (Figure 3) and exhibited higher sequence diversity. For the high peaks of N errors in Figure 3, a consistent sequential pattern was observed in both the mock community (Table 4) and pure cultures (Table 2), supporting the validity of the N correction algorithm. After the N correction method was applied to correct the Ns, the numbers of Ns from the mock community were significantly reduced, regardless of the different gene regions (Figure 3). Among reads, the reads with Ns represented 9.65% (16S), 9.58% (*bphD*) and 2.38% (*nifH*) (Figure 4). Among all the Ns, 86.54, 81.37 and 81.55% were corrected for 16S, *bphD* and *nifH*, respectively. Among all the corrected Ns matched with the reference sequences, 95.4% (16S), 96.9% (*bphD*) and 100% (*nifH*) were truly corrected, with small numbers of false corrections, indicating the high precision of the new N correction method. The N correction method decreased the overall rates of errors (Table 5).

**Figure 3.**
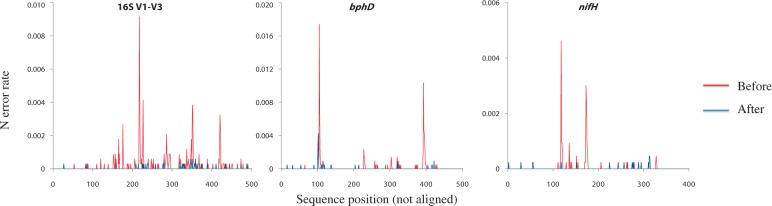
Distribution of Ns before (red line) and after (blue line) the N correction in the mock community. 16S, 16S V1–V3 region. *bphD*, *bphD* gene region. *nifH*, *nifH* gene region. The Y-axis unit is N error rate per base.

**Figure 4.**
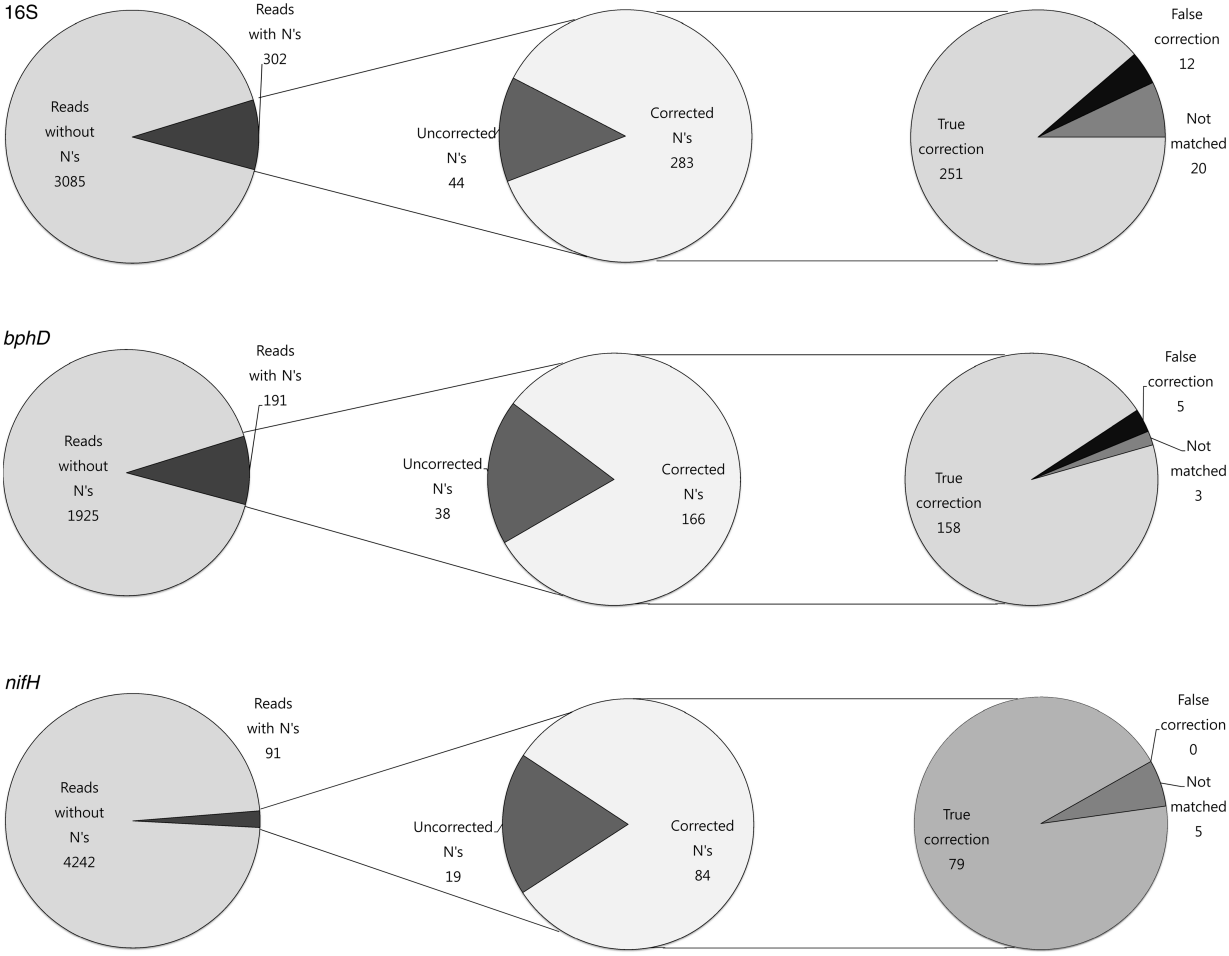
Precision analysis of the N correction using the mock community. 16S V1–V3, *bphD* and *nifH* indicate results from 16S V1–V3 region, *bphD* gene region and *nifH* gene region, respectively. The left pie graphs indicate the numbers of reads with and without Ns among the total reads. The intermediate pie graphs indicate the numbers of the corrected and uncorrected Ns among the total Ns. The right pie graphs indicate the numbers of truly and falsely corrected Ns among the corrected Ns. The ‘Not matched’ refers to Ns from the reads that were not matched with the reference sequences for the mock community.

**Table 4.** Sequence motifs of the highest N error rate areas from the mock community

Target region	Dominant base at N (true base)	Sequence motif of N formation
16S V1–V3	C	GGACCCGNGTCGCAT
*bphD*	C	CCCCCCANAGAAGGC
*nifH*	G	TGGGGGTNCGCTGTG

Homopolymers in front of the Ns are underlined.

**Table 5.** Comparison of error rates (average values) before and after N correction (from the mock community)

Error rate^a^ (per base)	16S V1–V3	16S V4–V5	*bphD*	*nifH*
Before length filtering and N correcting				
All reads	0.0147 (0.0234, 89%)	0.0059 (0.0110, 93%)	0.0085 (0.0135, 95%)	0.0062 (0.0112, 97%)
Reads with N	0.0235 (0.0286, 81%)	0.0105 (0.0116, 91%)	0.0134 (0.0129, 92%)	0.0161 (0.0142, 92%)
Reads without N	0.0138 (0.0226, 89%)	0.0058 (0.0112, 93%)	0.0080 (0.0135, 96%)	0.0060 (0.0110, 98%)
Reads with one N	0.0209 (0.0234, 84%)	0.0096 (0.0103, 94%)	0.0138 (0.0128, 93%)	0.0144 (0.0104, 95%)
Reads with two Ns	0.0516 (0.0472, 52%)	0.0171 (0.0169, 80%)	0.0140 (0.0133, 85%)	0.0202 (0.0159, 86%)
Reads with three Ns	0.0421 (N.A.)	0.0303 (0.0183, 25%)	N.A.^b^ (N.A.)	0.0419 (N.A.)
Before length filtering and after N correcting				
All reads	0.0145 (0.0233, 89%)	0.0058 (0.0110, 93%)	0.0083 (0.0135, 96%)	0.0061 (0.0111, 97%)
Reads with one correctable N	0.0194 (0.0262, 83%)	0.0077 (0.0096, 95%)	0.0110 (0.0128, 95%)	0.0096 (0.0076, 98%)
Reads with one uncorrectable N	0.0137 (0.0099, 92%)	0.0081 (0.0136, 94%)	0.0138 (0.0136, 86%)	0.0153 (0.0172, 81%)
Reads with more than one N (All Ns are correctable)	0.0478 (0.0432, 47%)	0.0117 (0.0136, 83%)	0.0108 (0.0092, 100%)	0.0173 (0.0132, 86%)
Reads with more than one N (Any Ns are uncorrectable)	0.0181 (N.A.)	0.0127 (0.0132, 91%)	0.0244 (0.0216, 67%)	0.0071 (N.A.)
After length filtering and N correcting				
All reads	0.0144 (0.0230, 89%)	0.0028 (0.0078, 98%)	0.0082 (0.0144, 95%)	0.0049 (0.0103, 99%)
Reads with one correctable N	0.0195 (0.0237, 82%)	0.0054 (0.0070, 99%)	0.0108 (0.0131, 95%)	0.0097 (0.0067, 100%)
Reads with one uncorrectable N	0.0135 (0.0090, 94%)	0.0051 (0.0050, 98%)	0.0113 (0.0040, 100%)	0.0093 (0.0048, 100%)
Reads with more than one N (All Ns are correctable)	0.0454 (0.0409, 47%)	0.0052 (0.0048, 100%)	0.0108 (0.0092, 100%)	0.0117 (0.0078, 100%)
Reads with more than one N (Any Ns are uncorrectable)	0.0181 (N.A.)	0.0150 (0.0144, 100%)	N.A. (N.A.)	0.0071 (N.A.)

The first and second values in each parenthesis represent (i) one standard deviation of error rates and (ii) the relative abundance of reads with error rates <3% in each read group.

^a^Error includes insertion, deletion, mismatch and N errors.

^b^N.A. stands for ‘not available’.

To examine the applicability of our N correction method to metagenomic data, the performance of our N correction method was also evaluated using previously reported metagenomic data (accession: SRX015617) from a mock community of 10 reference microbes in a pioneer study (20). Of 19 837 reads, 314 reads contained 333 Ns, representing a low N error rate per read (1.58%). Among all Ns, 215 Ns were corrected (64.56%). Among the corrected Ns matched with the reference sequences, 166 Ns were truly corrected (85.57%) (Supplementary Figure S6). The rates of N correction and true correction were slightly lower in these metagenomic data than in our amplicon data. These results demonstrate that N errors from metagenomic pyrosequencing are also correctable.

### N correction for analysis of an environmental microbial community

To determine the effect of removing reads with Ns and the correctability of Ns from an environmental sample, the amplicons of the 16S V1–V3 region from the direct extraction of DNA from the soil of Muak mountain were pyrosequenced. Of 3966 reads, 328 reads with 377 Ns were detected. When the Ns were corrected using our new method, 88.06% of the Ns (283 reads with 332 Ns) were incorporated in the N correction algorithm, demonstrating that the majority of Ns from the environmental sample were correctable (Supplementary Figure S7).

Depending on how to handle N errors, the results of microbial community composition analysis differed (Figure 5). After Ns were corrected using our method, the phylum-level microbial composition was almost identical with the original data; however, there was one more read from Firmicutes and one fewer from Proteobacteria. After all the N-containing reads were removed, the phylum-level microbial composition was slightly different from those of the original data and our N correction method. The portion of the phylum Bacteroidetes was significantly reduced after removal of the reads with Ns (chi-square = 11.6 with 1 df, *P* < 0.001). The result suggests that removal of the reads with Ns can cause a bias in microbial community structure analysis using 454 pyrosequencing.

**Figure 5.**
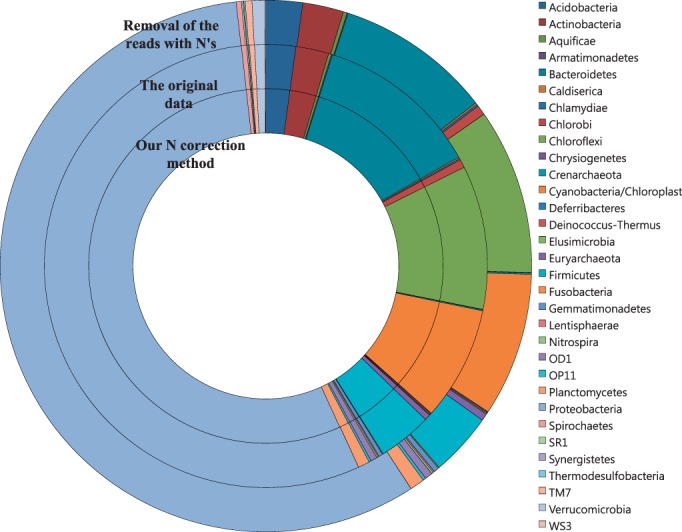
Comparison of the impacts of our N correction method and of the conventional method of removing N-containing reads (removal of reads with Ns) on soil bacterial community composition analysis. The original data indicate the soil bacterial community composition estimated from the 16S V1–V3 amplicon sequences before our N correction method or the removal of N-containing reads was applied.

### N error rates in response to homopolymer lengths

The effect of homopolymer length on the N error rate was determined for the pyrosequenced *bphD* amplicons from pure culture and the 16S amplicons from the soil microbial community (Supplementary Figure S8). Our sequence motifs with and without Ns were analyzed with respect to homopolymer length with chi-square tests, and N formation significantly differed depending on the homopolymer length both in pure culture (chi-square = 11 876 with 3 df, *P* < 0.001) and the environmental sample (chi-square = 5902 with 4 df, *P* < 0.001). The N error rates of the pure cultures increased with the lengths of the upstream homopolymers following a quadratic equation (*R*^2^ = 0.991). A consistent trend was observed for the results of the environmental microbial community (*R*^2^ = 0.998). These observations indicate that the presence of longer upstream homopolymers is associated with higher N error rates among reads, and this trend is applicable in settings ranging from pure culture to environmental samples.

### Error rate in response to the number of Ns and N correctability

To further evaluate the applicability of the N correction method, we examined error rates (per base) in response to (i) the number of Ns in a read and (ii) their N correctability. Before N correction and length filtration, the average error rates tend to increase with the number of Ns in a read (Table 5). Among the total reads with Ns, the relative abundances of the reads with one or two Ns were >97% (Supplementary Figure S9). Of the single N reads (the reads with one N), the average error rates were <3% (Table 5), and 84% (16S V1–V3), 94% (16S V4–V5), 93% (*bphD*) and 95% (*nifH*) had error rates <3%. Also, the average error rates of the reads with two Ns were <3% except 16S V1–V3, and this result is consistent with a recent quantitative study (26).

After the N correction was applied (without length filtration), among the N corrected single N reads, 83% (16S V1–V3), 95% (16S V4–V5), 95% (*bphD*) and 98% (*nifH*) had error rates <3%. The average error rates for the reads with one correctable Ns were lower than those for one uncorrectable Ns. One exception for this is the 16S V1–V3 result. Meanwhile, it is unclear whether the observed trend between correctable versus uncorrectable Ns is also true for the multiple N-containing reads, as the numbers of the multiple N reads with uncorrectable Ns were too limited (N = 1) for the 16S V1–V3 and *nifH* results. After the length filtering together with the N correction was applied, significant reduction in error rate was observed for the 16S V4–V5, *nif*H and *bph*D results (*t* test, *P* < 0.05) while not significant for the 16S V1–V3 result (*P* > 0.05), and 94% (16S V1–V3), 98% (16S V4–V5) 100% (*bphD*) and 100% (*nifH*) of the length-filtered reads with one uncorrectable N had error rates <3%, indicating an improved effect of the length filtering on sequencing accuracy.

## DISCUSSION

In this study, we demonstrated that most N errors from the 454 pyrosequencing system are sequence-dependent and correctable. If Ns occur in a random manner independent of the flanking sequences, then the Ns should be evenly distributed throughout the regions of the amplified genes. Even if the majority of Ns occur through the non-synchronized extension of fragments, then the majority of N errors should be located after certain positions. In fact, data from multi-templated beads may be filtered by sequencing companies, and most bioinformatics tools automatically remove long N homopolymer areas with low scores at the ends of reads. However, the results of the defined pure cultures and the mock community (Figures 1 and 3) allow us to rule out the possibility of the random formation of Ns by multi-templated beads and/or the non-synchronized extension of fragments. This finding is supported by the results from the environmental sample (Supplementary Figure S7). The presence of multi-templated beads and/or the non-synchronized extension of fragments was not observed, although the flow values and consensus of the aligned reads with Ns were investigated (Supplementary Figure S3). In addition, N formation was not significantly related to the hypervariable areas in 16S (27) or the wobble positions in the *bphD* and *nifH* regions (28). However, some of the uncorrectable Ns might have been attributed to the non-synchronized extension of fragments or multi-templated beads.

Although the exact cause of N formation is unknown, our observations clearly demonstrated that Ns frequently occur after their corresponding upstream homopolymers (Tables 2 and 4). The positive association between the length of the homopolymers and N error rates (Table 2 and Supplementary Figure S8) also indicates that homopolymers are an indirect cause of N errors. Homopolymer-dependent N errors may be due to a lack of available nucleotide reagents and/or the inhibitory effects of excessive by-products from the synthesis of its corresponding long upstream homopolymer. However, further evidence suggests that the inhibitory effects of by-products are a more likely cause. If a lack of nucleotide reagents *per se* is the major cause of N errors, a significant number of N errors must have occurred at the positions of the long homopolymers. Moreover, nucleotide reagents would not be insufficient for homopolymers ≤8 bp because the normalized light signal intensity is linearly proportional to the number of nucleotides for homopolymers of at least 8 bp (1). In addition, no significant evidence of the non-synchronized extension of fragments due to a lack of nucleotide reagents was found in the downstream areas around the positions of the N errors, suggesting that a lack of available nucleotide reagents *per se* may not be the major cause of N errors. In contrast, the excessive amount of by-products generated during the synthesis of upstream long homopolymers may have inhibitory effects on light emission but not synthesis. For example, pyrosequencing reactions produce 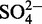 and oxyluciferin that inhibit the activities of ATP sulfurylase and luciferase, respectively (29). After the synthesis of upstream homopolymers, damaged nucleotide reagents may be produced as by-products that can be properly used for DNA synthesis, whereas the damaged nucleotide reagents release abnormal pyrophosphate (PPi) that cannot be converted to ATP in the next cycle of nucleotide reagent supply. Although an exact mechanism for base-specific N formation is unclear, we hypothesize that the damaged nucleotide reagents may have a base-specific inhibitory effect on the release of PPi from the next nucleotide of the same base, rather than causing the inhibition of DNA synthesis. Such base-specific inhibition may be transient, in that a certain portion of the inhibitory by-products accumulated during the upstream homopolymer synthesis may remain in the next cycle of the four nucleotide reagents (T, A, C, G), whereas no inhibitory by-products remain in the subsequent cycles of nucleotide reagent supply. A possible scheme for such base-specific and temporal N error events is presented in Figure 6. However, more detailed evidence is required to improve our understanding of the N errors of the pyrosequencing system. By-products may be one cause of other sequencing errors because N errors are associated with other types of errors.

**Figure 6.**
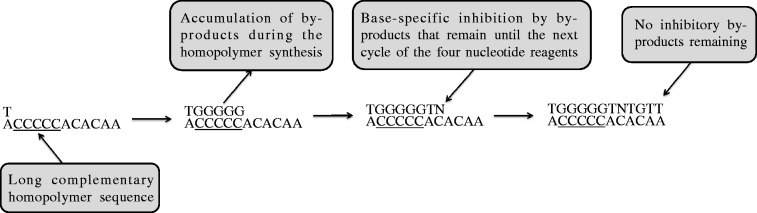
Possible causes of N formation. Some by-products from the homopolymer synthesis may be overproduced. They may remain up to the next cycle of the four nucleotide reagents (T, A, C, G) and have base-specific inhibition on the light emission of the same base.

Bioinformatics analysis of the reads with Ns and their flow value data from the pure cultures and mock community enabled us to identify the sequential pattern in front of the N, a homopolymer and 1–3 bases other the homopolymer in front of the N, and we observed that the true base of the following N is the same nucleotide as the upstream homopolymer. In addition, the observed trend was true not only for 16S but also for the functional genes, which strongly suggests that the majority of Ns are correctable in any gene-targeted pyrosequencing by replacing N with the base of the closest upstream homopolymer. To verify this concept, we developed a new algorithm to correct the Ns in the pyrosequenced amplicon reads and evaluated the correction performance of the new method. In the N correction algorithm, we corrected the Ns in the following order: (i) Ns for which a different base was present between the N and the upstream homopolymer, (ii) Ns for which two different bases were present between the N and the upstream homopolymer and (iii) Ns for which three different bases were present between the N and the upstream homopolymer. This order increased the precision of the algorithm in the 16S region because the closer homopolymers may have more significant effects on the formation of Ns (Figure 2). In the reverse order, the truly corrected Ns among all the corrected Ns matched with reference sequences were 87.8% (231/263, 16S), 97.5% (159/163, *bphD*) and 100% (79/79, *nifH*). For example, in TTCCGNA, N should be C based on our order, T in the reverse order. However, the true base for the N is C. The newly developed N correction algorithm demonstrates excellent N correction performance, but not all of the Ns were corrected, and false corrections were also detected (Figure 4). Some Ns arised regardless of the positions and homopolymers and remain uncorrected. One reason for false corrections may be the random formation of Ns. The majority of false corrections are triggered by short homopolymers, particularly those with a length of 2 bp. Another reason may be the effects of more distant homopolymers that are sometimes separated by more than one cycle of four nucleotide reagents. For example, in AAACCGGNGCTAATA, N should be C according to our algorithm, but A according to the reference sequences.

Our results demonstrate that rule induction and the algorithm for N correction are applicable for analysis of both 16S amplicons and functional gene-targeted amplicons. The functional gene amplicons from the mock community showed that the new method effectively corrected the majority of N errors with high precision. These results suggest that our N correction method is applicable to pyrosequencing reads of any genes. In addition, the rule induction and algorithm can be used to correct the Ns in metagenomic reads, as demonstrated by the results from the publicly available pyrosequencing metagenomic data (Supplementary Figure S6). However, the higher ratios of uncorrected Ns and false correction from the amplicon data suggest that our N correction method, which was developed for amplicon reads, can be further refined for metagenomic reads. Regardless, our findings suggest that our N correction method can be used for a wide range of pyrosequencing analyses to determine the exact bases of the Ns, particularly for difficult-to-sequence regions. The conclusions may be valid even in different pyrosequencing runs, as the different sequencing runs independently conducted for (i) the pure cultures, (ii) the mock community, (iii) the environmental sample and (iv) the public metagenome data resulted in the consistent observations of the dependence of the N errors on the sequential pattern and correctability of the N errors.

The effective N correction in the reads of the soil microbial community supports the applicability of the N correction method in the analysis of real environmental samples. The results of the sequence-dependent and correctable N formation have significant implications for microbial community and functionality analysis using gene-targeted pyrosequencing, which has been a popular tool for molecular microbial ecology and human microbiomes (30,31). For example, the N error rate of *P. naphthalenivorans* CJ2 (0.2657) was ∼20 times higher than that of *Rhodococcus* sp. RHA1 (0.0126) in the *bphD* region (Table 1). If we apply the current standard filtering method, the simple removal of all reads containing any N, the information for *P. naphthalenivorans* CJ2 would be more selectively discarded than that for *Rhodococcus* sp. RHA1. This suggests that the current N filtering method (simple removal of all reads with Ns) can cause a bias in microbial community composition analysis, as evidenced by the effect of the simple removal of reads with Ns on the soil microbial community structure (Figure 5).

The simple removal of all reads with N’s may be appropriate for the GS20 system, because high error rates (>5% per base) were observed in 16S amplicon reads with N’s (6). If the same is true for the newer 454 pyrosequencing system, our N correction would be useless because low-quality reads should be excluded for further community analysis. To test this possibility, we investigated the error rates in response to the numbers of Ns and their N correctability from the mock community (Table 5). The GS FLX and the GS20 systems provided a qualitatively similar trend of increasing error rates with increasing numbers of Ns in reads. However, the error rates were quantitatively different. In our study, most of the reads with Ns were single N reads (>90%), and most of the single N reads had error rates <3%. Even the average error rates of reads with two Ns were <3% except 16S V1–V3, which is coincident with a recent quantitative analysis for the GS FLX system (26). In addition, most of Ns in the single N reads were correctable (>70%), and the error rates of the correctable single N reads were lower than those of the uncorrectable single N reads. These suggest that a significant portion of Ns can be corrected by our N correction method, and the N-corrected single N reads had error rates <3%, which is appropriate for species-level analysis. Thus, these strongly support the usefulness of our N correction method. Nevertheless, the correctable single N reads still had higher error rates than the reads without N. This might be probably due to homopolymer-associated insertion/deletion errors in the reads with correctable Ns. Interestingly, the length filtering reduced the error rates of the reads with one uncorrectable N. The high error rates of the reads with the uncorrectable Ns (non-homopolymer-associated Ns) might have been attributed to non-synchronized extension of fragments or multi-templated beads in short reads. This suggests that the combined use of the N correction with length filtering can further improve the accuracy of the GS FLX pyrosequencing.

The N error occurrence in GS FLX can be grouped into three types. The first type Ns are associated with homopolymers, which are correctable by our N correction. The second type Ns are not associated with homopolymers but with read length, and may be attributed into non-synchronized extension of fragments or multi-templated beads in short reads. The error rate for the reads with the second type Ns can be improved by length filtering. The third type Ns are random formation due to uncertain factors. Additional studies on the third type Ns are needed to improve our understanding of N error. The findings from this work let us to propose a new procedure to use our N correction method in improving GS FLX sequencing quality, i.e. (i) filtering/denoising out low-quality reads based on flowgrams (30), quality score (QS) values (31) or/and read length (32), (ii) correcting the Ns associated with homopolymers and (iii) removing multiple N reads. In addition, it is recommendable to include amplicon(s) or genomic DNA from a defined mock community as a spike control in GS FLX runs for unknown environmental sample analysis, and to use the information from the mock community results in making decisions on (i) the cutoff level(s) for filtering out low-quality reads and (ii) if N-corrected multiple N reads have to be included or excluded in the following microbial community analysis. In conclusion, the findings support the appropriateness of our N correction method for the GS FLX system, and provide useful guidelines for improving the accuracy and precision of microbial community analysis using the GS FLX system.

## SUPPLEMENTARY DATA

Supplementary Data are available at NAR Online.

## FUNDING

“The GAIA Project” program of Korea Ministry of Environment. Funding for the open access charge: Korea Ministry of Environment, The GAIA Project.

*Conflict of interest statement*. None declared.

## Supplementary Material

Supplementary Data
