# Supplementary material for: Correction of sequence-dependent ambiguous bases (Ns) from the 454 pyrosequencing system
Source: Nucleic Acids Res. 2014 Jan 23;42(7):e51. doi: 10.1093/nar/gku070 (PMC3985643; doi:10.1093/nar/gku070)
Supplement: Supplementary Data [file supp_42_7_e51__index.html]

Correction of sequence-dependent ambiguous bases (Ns) from the 454 pyrosequencing system — Supplementary Data 

# Correction of sequence-dependent ambiguous bases (Ns) from the 454 pyrosequencing system

## Supplementary Data

files

**Files in this Data Supplement:**

- Supplementary Data - docx file
