# Supplementary material for: Correction of sequence-dependent ambiguous bases (Ns) from the 454 pyrosequencing system
Source: Nucleic Acids Res. 2014 Jan 23;42(7):e51. doi: 10.1093/nar/gku070 (PMC3985643; doi:10.1093/nar/gku070)
Supplement: Supplementary Data [file supp_gku070_nar-00508-met-k-2013-File010.docx]

Supplementary Table S1 | Genome sizes and gene copy numbers of the mock community.

| **Strain** | **Genome size**  **(bps)** | **Gene (# of gene copy)** |
| --- | --- | --- |
| *Bacillus cereus* ATCC 14579 | 5,427,083 | 16S (7) |
| *Burkholderia vietnamiensis* G4 | 8,391,070 | 16S (2), *nifH* (1) |
| *Burkholderia xenovorans* LB400 | 9,731,138 | 16S (2), *bphD* (1), *nifH* (1) |
| *Chromobacterium violaceum* ATCC 12472 | 4,751,080 | 16S (1) |
| *Corynebacterium glutamicum* ATCC 13032 | 3,282,708 | 16S (9) |
| *Desulfitobacterium hafniense* DCB-2 | 5,279,134 | 16S (5), *nifH* (4) |
| *Escherichia Coli* K-12 sub W3110 | 4,646,332 | 16S (6) |
| *Neisseria sicca* ATCC 29256 | 2,830,772 | 16S (1) |
| *Nostoc* PCC 7120 | 7,211,789 | 16S (2), *nifH* (3) |
| *Ochrobactrum anthropi* ATCC 49188 | 5,205,777 | 16S (1) |
| *Polaromonas naphthalenivorans* CJ2 | 5,366,143 | 16S (1), *bphD* (1), *nifH* (1) |
| *Pseudomonas pickettii* PKO1 | 5,325,729 | 16S (1) |
| *Pseudomonas putida* F1 | 5,959,964 | 16S (3), *bphD* (1), |
| *Rhodobacter sphaeroides* KD 131 | 4,711,139 | 16S (1), *nifH* (3) |
| *Rhodococcus sp.* RHA1 | 9,702,737 | 16S (1), *bphD* (6), |
| *Rhodospirillum rubrum* ATCC 11170 | 4,406,557 | 16S (1), *nifH* (5) |
| *Roseobacter denitrificans* Och 114 | 4,331,234 | 16S (1) |
| *Sphingobium yanoikuyae* B1 | 5,915,246 | 16S (1) |
| *Staphylococcus epidermidis* ATCC 12228 | 2,564,615 | 16S (1) |
| *Xanthomonas campestris* ATCC 33913 | 5,076,188 | 16S (1) |
| **Total : 20 strains** |  | **16S (48), *bphD* (9), *nifH* (18)** |


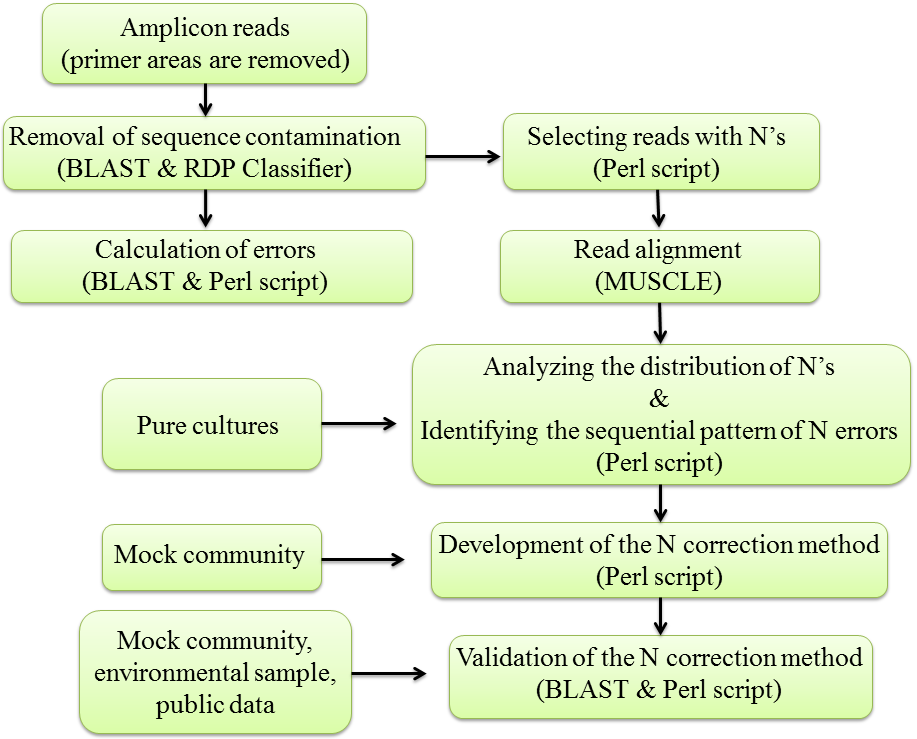


Supplementary Figure S2 | Generalized and simplified workflow of this study.


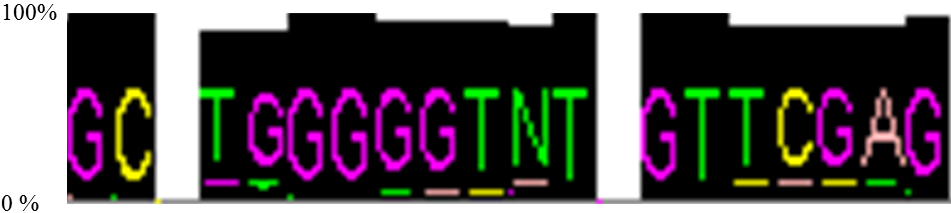


Supplementary Figure S3 | The consensus of aligned reads of Polaromonas_bphD in Table 2. Some parts are not graphically clear not because of multi-templated beads and/or non-synchronized extension of fragments within each sequence, but because of a few contaminant sequences.

Supplementary Table S4 | Data in Table 1. Some primer areas with few sequencing errors were deleted by mothur program. Damaged primer areas were manually deleted (Table 1). The primers of the *bphD* region were newly ordered.

PCR products No. of reads N error rate N error rate
 (per read) (per error*)

Staphylococcus _16S 2,723 0.0202 0.0282

Roseobacter_16S 4,178 0.0328 0.0219

Rhodococcus_16S 3,113 0.0630 0.0399

Polaromonas_bphD 1,645 0.2657 0.1566

Rhodococcus_bphD 1,194 0.0126 0.0158

Polaromonas_nifH 2,648 0.0076 0.0246

*Error includes insertion, deletion, mismatch, and N errors.

Supplementary Script S5 | Correction of reads with N’s. This script was coded in Perl.

open(RF, "<input.txt");

open(WF, ">output.txt");

while($line=<RF>)

{

if($line=~/^>/)

{

printf WF $line;

}

else

{

$line=~s/(AA[T]{1,4})N/$1A/g;

$line=~s/(TT[G]{1,4})N/$1T/g;

$line=~s/(GG[C]{1,4})N/$1G/g;

$line=~s/(CC[A]{1,4})N/$1C/g;

$line=~s/(AA[G]{1,4})N/$1A/g;

$line=~s/(TT[C]{1,4})N/$1T/g;

$line=~s/(GG[A]{1,4})N/$1G/g;

$line=~s/(CC[T]{1,4})N/$1C/g;

$line=~s/(AA[C]{1,4})N/$1A/g;

$line=~s/(TT[A]{1,4})N/$1T/g;

$line=~s/(GG[T]{1,4})N/$1G/g;

$line=~s/(CC[G]{1,4})N/$1C/g;

$line=~s/(AA[G]{1,4}[T]{1,4})N/$1A/g;

$line=~s/(AA[C]{1,4}[G]{1,4})N/$1A/g;

$line=~s/(AA[C]{1,4}[T]{1,4})N/$1A/g;

$line=~s/(TT[C]{1,4}[G]{1,4})N/$1T/g;

$line=~s/(TT[A]{1,4}[C]{1,4})N/$1T/g;

$line=~s/(TT[A]{1,4}[G]{1,4})N/$1T/g;

$line=~s/(GG[A]{1,4}[C]{1,4})N/$1G/g;

$line=~s/(GG[T]{1,4}[A]{1,4})N/$1G/g;

$line=~s/(GG[T]{1,4}[C]{1,4})N/$1G/g;

$line=~s/(CC[T]{1,4}[A]{1,4})N/$1C/g;

$line=~s/(CC[G]{1,4}[T]{1,4})N/$1C/g;

$line=~s/(CC[G]{1,4}[A]{1,4})N/$1C/g;

$line=~s/(AA[C]{1,4}[G]{1,4}[T]{1,4})N/$1A/g;

$line=~s/(TT[A]{1,4}[C]{1,4}[G]{1,4})N/$1T/g;

$line=~s/(GG[T]{1,4}[A]{1,4}[C]{1,4})N/$1G/g;

$line=~s/(CC[G]{1,4}[T]{1,4}[A]{1,4})N/$1C/g;

printf WF $line;

}

}

close(RF);

close(WF);


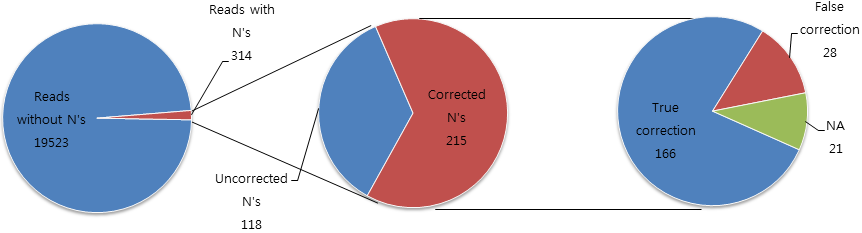


Supplementary Figure S6 | Precision analysis of the N correction using the public data (Accession: SRX015617) from a mock community (NA refers to the N-containing reads that were not matched with the reference sequences).


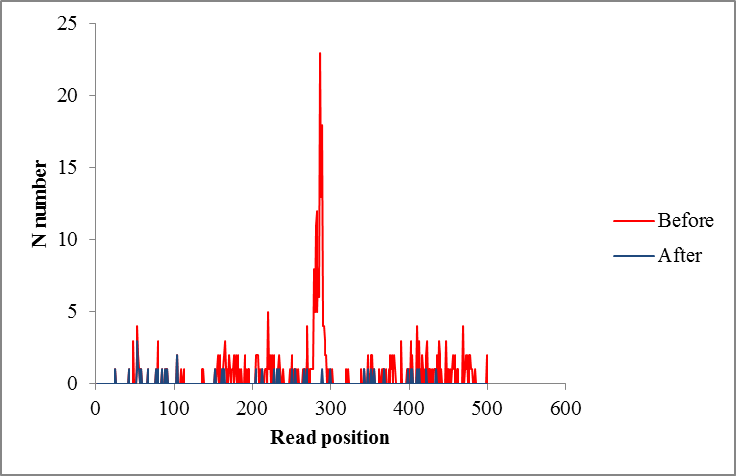


Supplementary Figure S7 | Distribution of N’s before and after the N correction in the soil sample. Before represents N number before applying our N correction method. After represents N numbers after applying our N correction method.


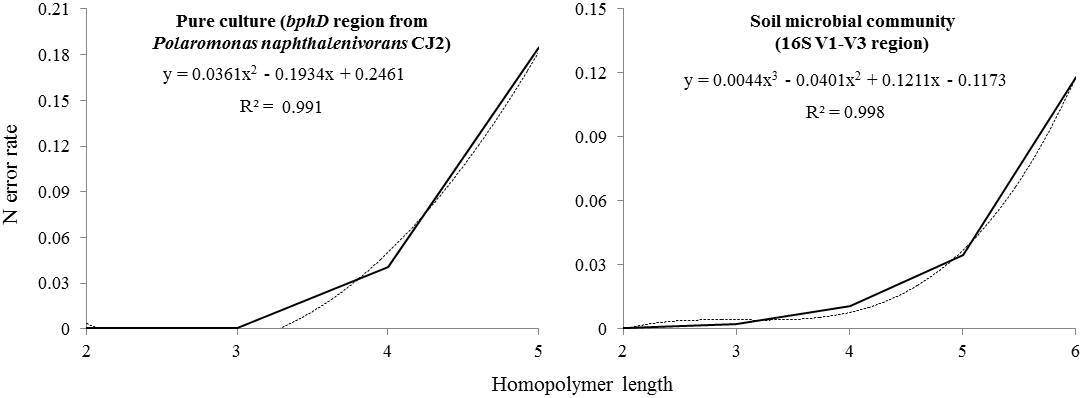


Supplementary Figure S8 | N error rates in response to the lengths of the upstream homopolymers. N error rates were analyzed in only areas that match the sequential pattern of N formation. The lengths of homopolymers correlate with the N error rates.

Supplementary Figure S9 | The percent of reads in response to the number of N’s. Amplicons of 16S V1-V3, 16S V4-V5, *bphD*, and *nifH* were sequences in the GS FLX system. The percents of reads separated by the numbers of N’s were calculated. Their overall error rates including substitution, insertion/deletion, and N errors were calculated. Azure, brown, yellowish green, and purple represent the percents of reads in response to the numbers of N’s (1, 2, 3, and 4, respectively).
